# Self-esteem related correlates of sleep quality in medical university students

**DOI:** 10.1192/j.eurpsy.2026.12100

**Published:** 2026-07-31

**Authors:** E. L. Nikolaev, S. A. Yusupov, N. D. Kodirov, J. N. Daminov

**Affiliations:** 1 Social and Clinical Psychology Department, I.N. Ulianov Chuvash State University, Cheboksary, Russian Federation; 2 Department of Pediatric Surgery No 1; 3 Department of Pharmacognosy and pharmaceutical technology; 4 Faculty of Pharmacy, Samarkand State Medical University, Samarkand, Uzbekistan

## Abstract

**Introduction:**

Researchers claim that the quality of sleep in medical students is subnormal, which may be due to a strenuous mental load they have while studying at university. How can such a highly stressful atmosphere influence a student’s self-esteem?

**Objectives:**

Our objective was to find out the interrelations of the sleep quality and sleep disorders with self-rated personal traits, states and behavior.

**Methods:**

When questioning 70 medical students of both gender aged 18-34, we used the Pittsburgh Sleep Quality Index (PSQI) questionnaire. We determined the students’ personal traits, states and behavior with the help of a self-rated questionnaire (Nikolaev, 2023), which evaluated the intensity of personal attributes on a 10-point scale. For mathematical treatment, we used the methods of descriptive statistics and correlational analysis.

**Results:**

The research revealed that two thirds of medical students (65.71%) experienced deterioration of their sleep quality. The mean score of the intensity of self-rated personal traits, states and behavior showed as follows: religious faith (8.79±1.90), happiness (8.34±2.18), confidence (8.07±2.27), successfulness (7.77±2.13), attractiveness (7.70±1.99), health (7.54±1.71), intelligence (6.99±2.15), well-being (6.86±2.61), physical fitness (5.56±2.49). PSQI global score in the surveyed students negatively correlates with their self-confidence (r=-.29; p<.05), happiness (r=-.25; p<.05), and health condition (r=-.24; p<.05). Sleep disturbance negatively correlates with confidence (r=-.30; p<.05) and attractiveness (r=-.28; p<.05). Poor sleep quality negatively correlates with the self-assessment of confidence (r=-.56; p<.05), happiness (r=-.37; p<.05), religious faith (r=-.29; p<.05), and frequency of physical activity (r=-.24; p<.05).

**Conclusions:**

Sleep quality in medical students correlates most of all with such a personal trait as confidence. However, it has weaker connections with the students’ perception of happiness and health. As sleep disorders may bring about deterioration of a personality’s psychological well-being, the revealed self-esteem related correlates might become a target for psychotherapists when working with university students.

**Disclosure of Interest:**

None Declared

